# Influence of Gelatin-Thrombin Matrix Tissue Sealant on Bacterial Colony Formation and Risk of Pelvic Infection

**DOI:** 10.1155/2016/2649708

**Published:** 2016-04-21

**Authors:** Michael J. Jarrett, Andres Vázquez-Torres, Daniel N. Frank, Bruce D. McCollister, Patrick K. Henthorn, Diana Ir, Jeanelle Sheeder, Michael S. Guy, Hiba Q. Anwar, Kian Behbakht

**Affiliations:** ^1^Research Track, University of Colorado School of Medicine, Aurora, CO 80045, USA; ^2^Department of Immunology and Microbiology, University of Colorado School of Medicine, Anschutz Medical Campus, Aurora, CO 80045, USA; ^3^Division of Infectious Diseases, Department of Medicine, University of Colorado School of Medicine, Anschutz Medical Campus, Aurora, CO 80045, USA; ^4^Division of Family Planning, Department of OB/GYN, University of Colorado School of Medicine, Anschutz Medical Campus, Aurora, CO 80045, USA; ^5^Division of Gynecologic Oncology, Department of OB/GYN, University of Colorado School of Medicine, Anschutz Medical Campus, Aurora, CO 80045, USA

## Abstract

*Objective*. Gelatin-thrombin matrix (GTM) tissue sealant use was previously identified as an independent predictor of pelvic infection following hysterectomies. We aim to elucidate contributing factors by assessing influence of GTM on bacterial colony formation and characterizing bacteria present at the vaginal cuff.* Methods*.* Escherichia coli* was incubated in phosphate-buffered saline (PBS) and pelvic washings with and without GTM to assess influence on colony formation. Pelvic washings of the vaginal cuff were collected from hysterectomies occurring from June through October 2015.* In vitro* techniques, 16S rRNA gene qPCR, and 16S amplicon sequencing were performed with washings to characterize bacteria at the vaginal cuff.* Results*. Mean bacterial colony formation in PBS was greater for* E. coli *incubated in the presence of GTM (1.48 × 10^7^ CFU/mL) versus without (9.95 × 10^5^ CFU/mL) following 20-hour incubation (*p* = 0.001). Out of 61 pelvic washings samples, 3 were culture positive (≥5000 CFU/mL) with* Enterococcus faecalis*.* Conclusion*.* In vitro* experiments support a facilitating role of GTM on colony formation of* E. coli* in PBS. However, given the negative results of surgical site washings following adequate disinfection, the role of GTM in promoting posthysterectomy pelvic infections may be limited. Analysis of pelvic washings revealed presence of* E. faecalis*, but results were inconclusive. Further studies are recommended.

## 1. Introduction

In a retrospective review gelatin-thrombin matrix (GTM) tissue sealant was identified as an independent predictor of pelvic abscess occurrence and pelvic infection following total hysterectomies [1]. GTM has been established as an effective means for controlling minor bleeding during surgical procedures [2, 3]. This hemostatic agent is used frequently during hysterectomy procedures due to its effectiveness and ease of use [1]. However, product insets that acknowledge GTM may increase rates of surgical site infections (SSIs) and may support bacterial proliferation [4]. GTM is composed of collagen and human thrombin [4]. We hypothesized these components may potentiate bacterial proliferation, thus contributing to increased rates of pelvic infection following hysterectomies.

Our first aim investigated the influence of GTM on bacterial colony formation in an environment free of other factors that may impact colony formation. To isolate the influence of GTM on colony formation* Escherichia coli* were incubated with and without GTM in phosphate-buffered saline (PBS), a solution that lacks substrates to support colony formation.* E. coli* is a pathogen frequently present in pelvic infections following hysterectomies and hence was used in experiments [5, 6]. Because PBS may not be representative of the microenvironment at the vaginal cuff, a second aim used washings of the vaginal cuff to assess influence of GTM on colony formation for bacteria present in washings. Quantitative polymerase chain reaction (qPCR) assays for 16S rRNA and gene sequencing of 16S rRNA amplicons provide a multifaceted analysis of bacteria in washings [7–9]. Due to low rates of colony formation in washings, a third aim inoculated washings with* E. coli* to assess influence of GTM in pelvic washings when bacteria are known to be present.

## 2. Materials and Methods

### 2.1. GTM Used in Experiments

Unused opened or unopened recently expired sterile GTM was collected for use in the protocol. Unused opened GTM was collected in sterile specimen cups within the sterile field in the operating room. These GTM samples were immediately transferred to the laboratory and stored in a −20°C freezer until use. Unopened GTM samples had expired within 3 months prior to use in experiments and were stored at room temperature until mixed for use. Aerobic cultures confirmed the sterility of GTM. GTM used in experiments was prepared by mixing 0.2 g of GTM in 12 mL of PBS. Mixtures were vortexed then incubated for 10–20 minutes at 37°C prior to use in experiments.

### 2.2. Pelvic Washings and Patient Data

#### 2.2.1. Study Population

Pelvic washings of the vaginal cuff were obtained from patients 18–85 years old who underwent hysterectomy procedures from June through October 2015. Washings were collected from abdominal and laparoscopic hysterectomy procedures including total abdominal, radical, laparoscopic, and laparoscopic assisted vaginal and robotic hysterectomies. Vaginal, supracervical, and cesarean hysterectomies were excluded. Nonhysterectomy procedures and procedures with active infection or immunosuppression were also excluded.

All patients received perioperative antibiotics (1 g Cefazolin IV) dosed thirty minutes to two hours prior to surgery and a 5-minute surgical site scrub was performed with a povidone-iodine preparation. In case of allergy to Cefazolin the alternative intravenous antibiotics used were 900 mg of clindamycin and 120 mg of gentamicin per standard of care. Providers did not routinely perform preoperative screening for bacterial vaginosis. The Colorado Multiple Institutional Review Board (COMIRB) approved the protocol as “not human subject research” and no patient consent was required.

#### 2.2.2. Collection of Pelvic Washings

Pelvic washings were performed with 0.9% saline following vaginal cuff closure. Approximately 50–100 mL of these washings was collected in sterile specimen cups. Although the washings were intended to be collected in sterile fashion within the sterile field, a large portion of washings were collected in vacuum containers and transferred into sterile specimen cups outside of the sterile field, but within the operating room. Sterile specimen cups containing pelvic washings were placed in sterile specimen bags and transported from the operating room to an administrative desk where they were stored in a cooler for approximately 0–8 hours until delivery to the laboratory. Deidentified patient information was collected in association with each washing sample. A nonaffiliated transportation team was utilized to move samples and patient data sheets from the operating room to an administrative desk to ensure patient data remained anonymous. Once in the laboratory, samples were placed in a 10°C refrigerator for up to 24 hours before processing.

#### 2.2.3. Sample Size

A study by Culligan et al. (2003) qualified 52% of vaginal surgical sites as “*contaminated*” (≥5000 CFU/mL) by 30 minutes after surgical scrub and 41% of cases by 90 minutes after scrub during vaginal hysterectomies. Based on these findings, we anticipated that 41–52% of pelvic washing collected following vaginal cuff closure during total hysterectomies would be “*culture positive*” (≥5000 CFU/mL) and 48–59% would be “*sterile*” (<5000 CFU/mL) [10]. We determined to collect pelvic washings from a minimum of 60 patients to provide an accurate representation of bacteria present at the surgical site following vaginal cuff closure.

### 2.3. Experiments

#### 2.3.1. Aims 1 and 3: Colony Formation of* E. coli* in PBS and Pelvic Washings

We compared growth of* E. coli* in PBS or sterile washings with and without GTM to assess influence on colony formation.* E. coli* stock solutions were prepared following incubation of the W3110 strain of* E. coli* in LB broth for 18–20 hours at 37°C. The average concentration of* E. coli* stock was determined following serial dilutions to be 3 × 10^9^ CFU/mL.* E. coli* stock mixtures were used to inoculate cultures of PBS or washings with and without GTM at a final concentration of 10^6^ CFU/mL. Cultures were incubated for 20 hours at 37°C in a shaker-incubator. Samples from each culture were taken at various time points during incubation, serially diluted in PBS, and plated in triplicate on LB agar. Agar plates were incubated for 18–20 hours and resultant colonies were counted to determine concentration of cultures in CFU/mL at each time point.

#### 2.3.2. Aim 2: Characterization of Bacteria Present at the Vaginal Cuff

*(1) Aerobic Cultures*. Aerobic cultures were performed to determine the influence of GTM on colony formation for bacteria present in pelvic washings. Mixtures of washings with and without GTM were prepared in triplicate and placed in a shake-incubator at 37°C for 18–20 hours. PBS alone and a mixture of GTM in PBS were incubated in similar fashion and used as controls. Mixtures were then serially diluted, plated on LB agar, and incubated for 18–20 hours at 37°C. Manual colony counts were used to determine concentrations of mixtures in CFU/mL. Initially, baseline cultures with pelvic washings at time-zero were not done prior to performing the experiment described above. When it became apparent this was missed, baseline cultures were performed using LB agar plates for the remainder of washing samples.

*(2) Gene Sequencing of Bacteria in Culture Positive Washings [11, 12]*. Gene sequencing of colonies produced from culture positive washings was done to identify the organisms present. Colonies produced on agar plates were collected and frozen at −80°C for genetic sequencing to be done at a later date. Sequencing of 16S rRNA amplicons was performed by the DNA sequencing and analysis service at Quintara (Denver, CO).

Nucleic acids were amplified using 16S rRNA gene primers SSU27F (AGAGTTTGATCCTGGCTCAG) and SSU1391R (GACGGGCGGTGWGTRCA: W, T, and R represent mixtures of nucleotides that increase the breadth of genes amplified in this process). Polymerase chain reactions (PCR) included 15 *μ*L of NovaTaq mastermix (Millipore Inc., Billerica, MA), 0.4 *μ*M of forward and reverse 16S targeted rRNA primers, and 2 *μ*L of purified oligonucleotides (30 *μ*L total volume). Thermocycler conditions were as follows: initial amplification at 94°C for 6 minutes, 30 cycles at 94°C for 30 sec, 53°C for 30 sec, 72°C for 1 min 20 sec, and a final extension at 72°C for 10 min, and then a hold at 4°C. PCR products were visualized via electrophoresis in a 2% agarose gel stained with ethidium bromide and directly placed into the zymo clean and concentrator (Zymo Research Inc., Irvine, CA). PCR products were diluted to 50 ng/*μ*L and quantified using Qubit Fluorometer 2.0 (Invitrogen, Carlsbad, CA). Paired-end sequences were assembled using the software phrap. Assembled sequences were compared with known sequences via a web-based BLAST search of the NIH GenBank nonredundant database.

*(3) qPCR Assays for 16S rRNA [11, 12]*. Centrifuged aliquots of pelvic washing samples were frozen at −80°C for use in PCR at a later date. PCR quantification of 16S rRNA was performed for a subset of 12 pelvic washing samples, including a mix of samples that produced bacterial colony formation* in vitro* and some that did not produce colonies.

After bead beating colonies on the Roche MagNA Lyser (Roche Inc., Basel, Switzerland), nucleic acids were extracted using the PowerViral Environmental RNA/DNA Isolation kit (Mobio Inc., Carlsbad, CA). In order to detect 16S rRNA, the extracted nucleic acids were used as a template for qPCR using specific bacterial-targeted universal 16S total bacterial primers and a 6-carboxyfluorescein-labeled TaqMan Probe on the Biorad CFX96 instrument (Biorad Inc., Hercules, CA). PCR reactions included 10 *μ*L of Dynamo ColorFlash PCR mastermix (Thermoscientific Inc., Waltham, MA), 0.2 *μ*M of TaqMan 16S total bacterial primer oligonucleotides, and 2 *μ*L of purified target nucleic acids (20 *μ*L total volume). The qPCR conditions began with an amplification at 95°C for 10 min, followed by 40 cycles at 95°C for 15 sec and 60°C for 1 min 30 sec.

### 2.4. End Points and Data Analysis

The continuous variable for Aims 1 and 3 was average bacterial concentration in CFU per mL, calculated from mean aerobic bacterial colony counts. Average concentrations of bacteria in CFUs per mL were compared across groups and time points using paired *t*-tests and repeated measures ANOVA with Bonferroni post hoc tests. Descriptive statistics were performed using IBM SPSS version 23.

Endpoints for* in vitro* experiments in Aim 2 include two categorical variables. Pelvic washings that produced <5000 CFU/mL via aerobic cultures following 20-hour incubation were categorized as “*sterile*” [10]. Washings that produced bacterial concentrations ≥5000 CFU/mL following 20-hour incubation were categorized as “*culture positive*” [10].

The continuous variable in the third aim was the cycle threshold (Ct) value, defined as the number of cycles required for a fluorescent signal to cross the threshold value and thus exceed background level during qPCR assays for 16S rRNA [11]. Ct values < 25 are very strong positive reactions indicative of abundant 16S rRNA in a sample; values of 25–29 reflect moderately strong positive reactions; values of 30–35 are positive reactions; 36 value indicates weak reactions with minimal target nucleic acid present; and values of 37–40 are negative reactions, indicating absence of target nucleic acid in samples [11].

## 3. Results

### 3.1. Aims 1 and 3: Colony Formation of* E. coli* in PBS and Pelvic Washings

Mean bacterial colony formation after 20-hour incubation in PBS was greater for* E. coli* with GTM versus without (1.48 × 10^7^ CFU/mL versus 9.9 × 10^5^ CFU/mL, *p* = 0.001). There was no difference in concentration of* E. coli* in PBS with and without GTM at time-zero (7.9 × 10^6^ CFU/mL versus 6.7 × 10^5^ CFU/mL, *p* = 1.00). Mean concentrations of* E. coli* in PBS with and without GTM following 20-hour incubation were 1.48 × 10^7^ CFU/mL ± 3.56 × 10^6^ CFU/mL and 9.9 × 10^5^ CFU/mL ± 3.5 × 10^5^ CFU/mL, respectively.

There was no change in concentration of* E. coli* following 20-hour incubation in pelvic washings with GTM (4.3 × 10^5^ CFU/mL, time-zero versus 4.46 × 10^6^ CFU/mL – 20 hours; *p* = 0.12) and without (4.3 × 10^5^ CFU/mL, time-zero versus 8.29 × 10^6^ CFU/mL – 20 hours; *p* = 0.06). Mean concentrations of* E. coli* in pelvic washings with and without GTM following 20-hour incubation were 4.46 × 10^6^ ± 7.14 × 10^6^ CFU/mL and 8.29 × 10^6^ ± 1.08 × 10^7^ CFU/mL, respectively.

**Figure 1:**
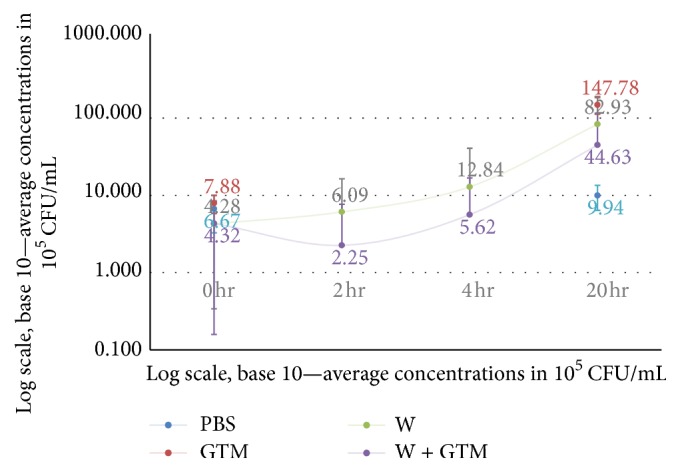
Colony formation of* E. coli* in PBS or pelvic washings ± GTM. PBS: phosphate-buffered saline. GTM: gelatin-thrombin matrix tissue sealant. W: pelvic washing.

Fifty-six percent of cultures of* E. coli* in pelvic washings + GTM (*n* = 5/9) and thirty-three percent of cultures without GTM (*n* = 3/9) produced no colony formation following 20-hour incubation. There was no difference in colony formation between cultures of* E. coli* in washings with versus without GTM at 2 hrs (2.3 × 10^5^ CFU/mL versus 6.1 × 10^5^ CFU/mL, *p* = 0.33) and 4 hrs (5.6 × 10^5^ CFU/mL versus 1.28 × 10^6^ CFU/mL, *p* = 0.48). Mean colony formation following 20-hour incubation was greater in PBS with GTM versus pelvic washings with GTM (1.48 × 10^7^ CFU/mL versus 4.46 × 10^6^ CFU/mL, *p* = 0.016). Results described above for Aims 1 and 3 are represented in Table 1 and Figure 1.

### 3.2. Aim 2: Characterization of Bacteria Present at the Vaginal Cuff

#### 3.2.1. Aerobic Cultures and Patient Data

Pelvic washings and patient data were collected from a total of 61 patients, ages 29 to 83 years old (mean = 51.2 ± 12.3 yrs). Two culture positive washings displayed colony formation with and without GTM; a third culture positive washing only produced colonies when incubated without GTM. Baseline cultures at time-zero were sterile for two of three culture positive washings and not performed for the third. The remaining 58 washing samples incubated with and without GTM displayed no colony formation and were categorized as sterile. One washing sample was culture positive in time-zero cultures yet it displayed no colony formation when incubated with and without GTM.

Two of three culture positive washings came from patients who had a preoperative diagnosis of endometrial cancer and history of appendectomy. A diagnosis of cancer was present in 29 of 61 patients, the most common being endometrial cancer (*n* = 17), followed by ovarian (*n* = 5), and cervical cancer or dysplasia (*n* = 4). A large proportion of patients had persistent uterine bleeding (*n* = 10), fibroids (*n* = 9), or endometriosis (*n* = 6) as an indication for surgery. The majority of procedures were total abdominal and robotic hysterectomies (*n* = 32 and *n* = 17). Total laparoscopic (*n* = 7), radical (*n* = 3), and laparoscopic assisted vaginal hysterectomies (*n* = 2) were in the minority. Two patients had a history of pelvic infection and twenty-six patients had a history of pelvic operation, the most common being cesarean section (*n* = 9 patients). The surgeons' intraoperative impressions noted pelvic adhesions in 10 patients, most of whom had a history pelvic operation (*n* = 9).

#### 3.2.2. Gene Sequencing of Bacteria in Culture Positive Washings

Sequencing of 16S rRNA amplicons from colonies produced by all three culture positive pelvic washings revealed presence of* Enterococcus faecalis*. One culture positive washing sample also contained an uncultured* Corynebacterium *species.

#### 3.2.3. qPCR Assays for 16S rRNA

The results of qPCR experiments for the subset pelvic washings showed Ct values for all 12 washings were > 37. These results did not differ from those obtained from reagent-only controls indicating that any bacteria present in the washings were below the detection limits of the qPCR assay.

## 4. Discussion

Gelatin-thrombin matrix tissue sealant is an effective hemostatic agent useful in many operative scenarios [2, 3, 13]. As referenced in Introduction, GTM was previously identified as an independent predictor of pelvic abscess following total hysterectomies [1]. The study by Anderson et al. (2014) found that pelvic abscesses occurred in 11 patients, 9 of whom received GTM (82%). All patients in this study by Anderson et al. received the same preoperative antibiotics and surgical site scrub that were utilized for patients included in our study.* In vitro* experiments in our study may support a facilitating role of GTM on colony formation of* E. coli* in PBS. However, given the negative results of surgical site washings following adequate disinfection, the role of GTM in promoting postoperative pelvic infections may be limited. Future studies may include a similar experiment involving addition of a broad-spectrum antibiotic to GTM to assess whether colony formation of* E. coli* in PBS can be reduced or eliminated.

As seen in the paper by Culligan et al. (2003), viable bacteria are present within the vagina up to 90 minutes following surgical scrub during vaginal hysterectomies. These bacteria are likely present at the vaginal cuff, albeit in lower concentration than within the vagina based on our findings. We anticipated that 41–52% of pelvic washings collected (*n* = 25–32) following vaginal cuff closure would be culture positive and 48–59% (*n* = 29–36) would be sterile [10]. In our study 5% (*n* = 3/61) of washings were culture positive. Culligan et al. (2003) acknowledge preoperative antibiotics and surgical site scrub likely accounted for the progressive decline in bacterial colony formation observed throughout the duration of the procedures. In our study, perioperative antibiotics and surgical site scrub also likely account for the unexpected low rate of culture positive washings and variable growth of* E. coli* in pelvic washings. Further, baseline cultures missed for a proportion of samples may have limited our ability to culture bacteria from washings.

Other factors may account for the unexpected low percentage of culture positive washings in our study. Culligan et al. (2003) performed anaerobic cultures as part of their protocol, while we did not due to resources prioritization. Aerobic cultures, qPCR, and gene sequencing were initially deemed adequate to provide comprehensive characterization of bacteria present in washings. In hindsight, due to limitations encountered in qPCR experiments, anaerobic cultures would have been beneficial to include. Culligan et al. (2003) collected vaginal swabs using an aerobic/anaerobic transport system and cultures were performed immediately following transport to the laboratory. We collected washings of the vaginal cuff performed with 0.9% saline, transported samples in sterile specimen containers, and refrigerated samples for up to 24 hours prior to performing cultures. Washing samples were refrigerated and processed the following day as a batch to make the protocol practical for laboratory staff to perform within normal working hours. Accordingly, a proportion of viable bacteria in washings were likely lost during transport and storage. Further, our surgical teams often used more than 200 mL while performing pelvic washings, thus diluting bacteria that may have been present at the surgical site.

All three* culture positive* washing samples produced* E. faecalis*, an organism endogenous to the large intestine and a component of vaginal flora [6, 11].* E. faecalis* is a dominant pathogen involved in pelvic abscesses [6, 14]. An uncultured* Corynebacterium *sp. presents in one culture positive sample and is also endogenous to the vagina, other mucus membranes such as the oropharynx, and can be found on skin surfaces such as the hands [6, 15]. This organism has been identified as a pathogen involved in pelvic infections as well [14, 15].

It is possible these organisms were present as a result of contamination during experiments, as baseline cultures were negative for two of three culture positive samples and not performed for the third. However,* Staphylococcus epidermidis* is the organism most predominant on hands and one would expect that this species would be present if lab staff contaminated samples [15]. With all three culture positive samples producing* E. faecalis*, it seems more likely that this organism was present at the vaginal cuff following incision through the vagina. It is possible* E. faecalis* was present in washings at a low concentration and thus missed by baseline cultures. Further, if washings indeed contained perioperative antibiotics and surgical scrub prep, it is possible* E. faecalis* could gain resistance to antibiotics and produce colonies over time. To provide further insight, pelvic washings could be analyzed for presence of antibiotics and surgical scrub prep. Additionally, whole gene sequencing could be performed to determine whether* E. faecalis* came from the same person (i.e., lab staff) as a result of contamination during experiments or if the bacteria came from the different individuals, suggesting contamination of the surgical site with vaginal flora.

For 2 of 3* culture positive* washings, qPCR for 16S rRNA found Ct values > 37, interpreted as negligible or no biomass present. Assays for the third culture positive sample were unsuccessful due to inhibition of fluorescence by blood in washings. Although the results of qPCR assays suggested absence of bacteria in washings, small volumes (500 *μ*L) of washings are used for qPCR experiments and thus viable bacteria may have been missed. However, with culture positive washings producing ≥5000 CFU/mL, it seems qPCR should have detected target nucleic acids in spite of small volumes used. Overall, results from qPCR assays are inconclusive.

## 5. Conclusions

*In vitro* experiments support a facilitating role of GTM on colony formation of* E. coli* in PBS. However, given the negative results of surgical site washings following adequate disinfection, the role of GTM in promoting posthysterectomy pelvic infections may be limited. Analysis of pelvic washings revealed presence of* E. faecalis*, but results were inconclusive. Further studies are recommended. GTM should be used judiciously and only according to manufacturers' instructions to minimize risk of infection.

## Figures and Tables

**Table 1:** Colony formation of *E. coli* in PBS or pelvic washings ± GTM.

Time	Sample	*n*	Mean colony formation (10^5^ CFU/mL)	Std. deviation	95% confidence interval for mean		Min.	Max.	Sig. (*p*)
					Lower bound	Upper bound			
0 hrs	PBS	9	6.7	3.4	4.1	9.3	1.5	11.0	1.000
	GTM	8	7.9	2.1	6.1	9.6	3.5	10.0	
	W	9	4.3	3.9	1.3	7.3	0.0	9.0	
	W + GTM	9	4.3	4.2	1.1	7.5	0.0	10.5	

2 hrs	PBS								
	GTM								
	W	9	6.1	10.2	−1.7	13.9	0.0	29.5	
	W + GTM	9	2.3	5.4	−1.9	6.4	0.0	16.5	

4 hrs	PBS								
	GTM								
	W	9	12.8	27.7	−8.5	34.1	0.0	80.0	
	W + GTM	9	5.6	11.2	−3.0	14.2	0.0	27.0	

20 hrs	PBS	9	9.9	3.5	7.2	12.7	6.5	16.0	0.001
	GTM	9	147.8	35.6	120.4	175.2	70.0	195.0	
	W	9	82.9	107.8	0.1	165.8	0.0	325.0	
	W + GTM	9	44.6	71.4	−10.3	99.5	0.0	175.0	

PBS: phosphate-buffered saline.

GTM: gelatin-thrombin matrix tissue sealant.

W: pelvic washings.
